# Navigated Transcranial Magnetic Stimulation: A Biologically Based Assay of Lower Extremity Impairment and Gait Velocity

**DOI:** 10.1155/2017/6971206

**Published:** 2017-01-24

**Authors:** Heather T. Peters, Kari Dunning, Samir Belagaje, Brett M. Kissela, Jun Ying, Jarmo Laine, Stephen J. Page

**Affiliations:** ^1^Division of Occupational Therapy, The Ohio State University, Columbus, OH, USA; ^2^B.R.A.I.N. (Better Rehabilitation and Assessment for Improved Neuro-recovery) Laboratory, Ohio State University, Columbus, OH, USA; ^3^The University of Cincinnati, Cincinnati, OH, USA; ^4^Department of Neurology, Emory University School of Medicine, Atlanta, GA, USA; ^5^Nexstim, Ltd., Helsinki, Finland

## Abstract

*Objectives*. (a) To determine associations among motor evoked potential (MEP) amplitude, MEP latency, lower extremity (LE) impairment, and gait velocity and (b) determine the association between the presence of a detectable MEP signal with LE impairment and with gait velocity.* Method*. 35 subjects with chronic, stable LE hemiparesis were undergone TMS, the LE section of the Fugl-Meyer Impairment Scale (LE FM), and 10-meter walk test. We recorded presence, amplitude, and latency of MEPs in the affected tibialis anterior (TA) and soleus (SO).* Results*. MEP presence was associated with higher LEFM scores in both the TA and SO. MEP latency was larger in subjects with lower LEFM and difficulty walking.* Conclusion*. MEP latency appears to be an indicator of LE impairment and gait.* Significance*. Our results support the precept of using TMS, particularly MEP latency, as an adjunctive LE outcome measurement and prognostic technique.

## 1. Introduction

Two-thirds of the growing stroke survivor population exhibits significantly diminished walking ability [1, 2], making walking retraining a major focus of stroke rehabilitation [3, 4]. Precise measurement of deficits is fundamental to characterizing patients' impairment and to planning cost effective, appropriate, lower extremity (LE) interventions [5]. Consequently, a variety of behavioral measures are deployed to quantify paretic LE outcomes [6–8].

Clinical assessment tools (e.g., Timed Up and Go and Dynamic Gait Index) provide clinicians with valuable insight into patients' ambulatory independence, which greatly influences the course and content of rehabilitative therapies. However, outcomes from such performance-based assessments are associated with extraneous (e.g., fear of falling [9, 10]) and/or peripheral variables (e.g., osteoarthritis [11]; diminished cardiorespiratory fitness [12, 13]), which can raise the likelihood of Type I and Type II errors. These instruments are also limited in that they are subjective and do not provide direct insight into central nervous system (CNS) response to restorative approaches, that is, a limitation, given that insufficient activation of the LE musculature is the primary impairment underlying walking deficits after stroke [14].

Volitional ambulation is activated by neural impulses travelling primarily via the corticospinal tract (CST) [15]. These descending CST pathways transmit signals primarily to the contralateral extremities, with a small percentage of signals transmitted ipsilaterally [16]. Poststroke motor evoked potentials (MEPs) reflect excitability of the lesioned areas, alterations in interhemispheric communication, and resultant CST activity [17]. The presence of MEPs from upper extremity muscles is associated with a more favorable prognosis after stroke [18, 19], while MEP amplitude is correlated with upper extremity impairment [20]. MEPs have likewise been advocated as a biological method for measuring and predicting poststroke ambulation changes [21–23]. However, associations between MEPs and LE outcomes have not been investigated in stroke, aside from one case report during the acute phase [18, 20, 24], when considerable spontaneous recovery and multiple interventions are cooccurring.

Given the increasing prevalence of stroke survivors and diminishing length of stays in rehabilitative settings, assessment methods to direct LE treatment must be optimized. Could MEPs constitute a biologically based, objective, method to address this need? Our overall objective was to examine the association of MEPs with LE outcomes in a well-defined cohort of chronic, stable, stroke survivors. To accomplish this objective, the study had two primary aims: (1) to determine associations among tibialis anterior (TA) and soleus (SO) MEP characteristics (amplitude and latency) and scores on clinical measures; (2) to determine associations among the presence of a detectable MEP signal and scores on clinical measures. Within these two primary aims, we specifically examined the following associations: (a) TA and SO MEP amplitude/latency and scores on the LE FM; (b) TA and SO MEP amplitude/latency and gait; (c) presence of a MEP response and LE FM scores; and (d) presence of a MEP response and gait. To our knowledge, this was the first study to extensively examine MEPs as a measure of LE outcomes after stroke, as well as being one of the first studies to use navigated TMS in either the paretic upper or lower extremities to associate MEP presence with outcomes.

## 2. Methods

### 2.1. Subjects

Volunteers were recruited directly from local stroke support groups and by using advertisements placed in local outpatient stroke clinics. After signing an approved consent form, the following study criteria were applied to volunteers expressing interest in the study:* Inclusion criteria* were as follows: (a) ≥20 years of age; (b) unilateral stroke experienced ≥ 4 months prior to study enrollment, occurring in middle cerebral artery (MCA) territory involving the motor cortex (cortical stroke) or/and its corticospinal projections (subcortical stroke); (c) no other known brain abnormalities by history or by structural MRI.* Exclusion criteria* were as follows: (a) contraindications to neuroimaging as described in detail elsewhere [25] (e.g., seizure history; pregnancy; metal in head; implanted medical devices); (b) history of alcohol abuse and/or drug use; (c) history of mental illness; (d) personal or family history of epilepsy; (e) hypertensive or hypotensive condition; (f) any condition that would prevent the subject from giving voluntary informed consent; (g) taking any medication that interferes with the TMS measures; (h) enrolled in an interventional trial during this study; (i) a fixed contraction deformity in the paretic LE; (j) excessive spasticity in any joint of the affected LE as indicated by the Modified Ashworth Spasticity (MAS) Scale ≥ 2.

### 2.2. Instruments

Given that LE impairment was the primary study outcome, the primary outcome measure was the LE section of the* Fugl-Meyer Impairment Scale (FM)* [6]. The FM has a maximum score of 34, with individual items examining paretic LE reflexes, isolated movement at joints in the paretic LE, and speed of movement. Thus, the FM enabled our team to examine the influence of the CST on LE active movement in an iterative, quantified, way.

We also wished to determine whether isolated movements activated primarily by the CST affected functional outcomes. Gait velocity is a reliable, valid, and sensitive measure of poststroke mobility and function [26] that is highly correlated with recovery and independence [27]. Thus, we measured gait velocity during a* 10-meter walk test*, administered at both a self-selected and fast speed. The 10-meter walk test is a commonly used measure of gait velocity and has shown excellent reliability [28] and validity [29] in the poststroke population. Gait velocity was assessed in a subset of our sample (*n* = 26) that could ambulate safely without use of an ankle foot orthosis (AFO) or adaptive equipment (e.g., a cane or walker), enabling more pure assessment of the association between CST integrity and ambulation without the mitigating impact of extraneous assistance. Difficulty to walk was a binary variable defined as those subjects who reported being unable to walk without AFO or had self-selected gait speed <60 cm/sec or had fast gait speed <80 cm/sec.

### 2.3. Testing Procedures

**Figure 1:**
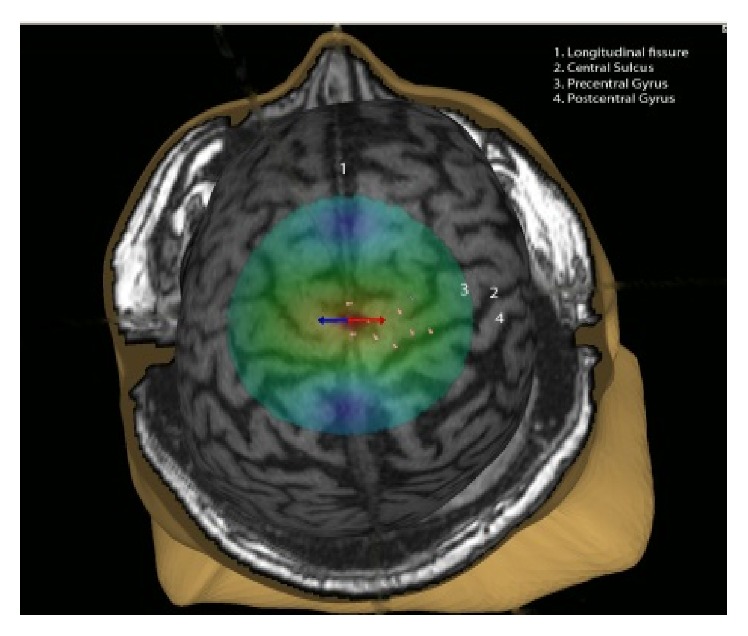
Example image obtained during stimulation of the paretic lower extremity cortical areas.

To obtain MEPs, transcranial magnetic stimulation (TMS) was used. Conventionally, TMS has consisted of applying an electromagnetic field to a particular cortical area believed to control a certain function (for a review see ref. [30]). However, a common challenge associated with conventional TMS is identifying the proper stimulation site on the cortex, as one must do so based on landmarks on the head and estimation of normal brain topography. Unlike the upper extremity representation, LE cortical representations are buried deep in the junction of central sulcus and longitudinal fissure and the variability of individual cortical gyri is considerable. Since the distance from coil to target LE representation is also larger than that of hand motor representations, optimal stimulation of LE cortex may also be challenging to estimate, in particular in patients with compromised function due to lesions such as stroke. Navigated brain stimulation (NBS) integrates a particular patient's brain MRI into his/her stimulation procedures. The MRI essentially acts as a “map,” enabling real-time location of where the magnetic coil is located and the area being stimulated (Figure 1).

In the current study (and consistent with the above), a high-resolution 3-dimensional, T1 weighted MRI was first obtained for each subject's brain to use with the navigation system (Nexstim eXimia). Next, we performed each subject's brain to head coregistration by identifying 3 landmarks on the MRI (the tragus of the right and left ears; the bridge of the nose) and marking them on the subject's head using a digitizing stylus.

TMS was applied through a figure-of-eight coil that was 70 mm in diameter (eXimia 3.2. stimulator, Helsinki, Finland). The motor threshold (MT) was defined as the lowest stimulation strength (in V/m) and in stimulator output (%) to produce a response of greater than 50 *μ*V in the paretic abductor pollicis brevis muscle (APB) and to locate the central sulcus functionally. This information was used as a basis for LE mapping. Specifically, the TA and SO located near the central fissure were identified on each subject's brain and stimulated first with 110% of APB MT +20 V/m stimulation intensity at rest. This intensity was chosen as a starting point as it has been shown to be sufficient to elicit LE MEPs in healthy subjects [31]. Coil orientation was based on previous work by Groppa and colleagues [21] stating that coil placement should be perpendicular to the longitudinal fissure at the junction of central sulcus and central fissure with the coil angled to induce coronally oriented left-to-right current flow for the right LE or right-to-left current flow for the left LE. Stimulation was continued by following the longitudinal fissure 2 cm anteriorly and 2 cm posteriorly in steps with 2-3 mm spacing. Stimulation was also performed perpendicular to the uppermost part of central sulcus, 3 cm from the longitudinal fissure. If there was a positive muscle response during any of these attempts, the intensity was lowered −10 V/m until the response was 100–600 uV and the muscle (TA or SO) was mapped. If there was no response, the subject was asked to actively move the muscle (TA or SO). Intensity was increased in steps of +10 V/m until a 100–600 uV response was obtained or the maximum output of the stimulator was reached and the muscle (TA or SO) was mapped.

Presence of MEP responses in TA and SO muscles was tested and recorded for all locations. When the stimulation of the primary motor cortex (M1) of the affected hemisphere did not elicit a discernible, reproducible, MEP amplitude in at least five out of ten stimulations at any location, this was considered “no response” (coded as MEP response = 0). When the stimulation of the M1 of the affected hemisphere at a specific location elicited a discernible MEP amplitude in at least five out of ten stimulations, it was considered a “response” (coded as MEP response = 1). The latency was defined as the time from the onset of stimulus to the onset of MEP. For subjects with a MEP response, the peak-to-peak amplitude was measured. The average of amplitudes (or latencies) from observed trials was obtained as the outcome measure for each subject with response to a targeted TA (or SO) muscle. As an alternative method, the maximum of amplitudes (or its corresponded latency) was also considered as the outcome measure for the study.

### 2.4. Data Analyses

The binary measure of MEP response was compared of rates between affected side and unaffected side using McNemar's test. For numerical outcome measures of amplitude (AMP) and latency (LAT), they were log-transformed to correct right skewness before formal analysis. Log-transformed variables (called Ln_AMP and LN_LAT) were then compared between affected and healthy sides using a mixed effect model, after correcting for within person correlation using a random effect. For the affected TA and SO, mean LEFM score was compared between subjects with and without MEP responses using a two-sample *t*-test, and the rates of difficulty to walk were compared between groups using a Chi-square test. The Ln_AMP and Ln_LAT were assessed of their relationships to LEFM score using Pearson's correlation coefficients and compared of means between subjects with and without having difficulty to walk using two-sample *t*-tests.

The aforementioned unadjusted analyses were then repeated using multivariate mixed effect models to investigate the between-group means after adjusting for controlling covariates, such as age, gender, and duration of stroke. Results from both unadjusted and adjusted analyses were reported in this paper. The study also provided analyses on AMP and LAT measures using the maximum methods. Those results were not reported as the findings were consistent to the current (averaged AMP and LAT) method.

All statistical methods were performed using SAS 9.4 software (SAS, Cary, NC). *p* values < 0.05 were considered statistically significant.

## 3. Results

### 3.1. Subject Demographics

Using the aforementioned study criteria, 35 subjects were included (demographics depicted in Table 1).

### 3.2. Outcomes

As shown in Table 2, while the TMS input parameters stimulation intensity and electronic field (EF) were the same between affected and unaffected sides, MEP response rates were different. The affected side had significantly lower response compared to the unaffected side for both TA and SO. Among subjects with MEP responses, latency was longer (larger) in the affected side versus the unaffected side.

Table 3 shows that subjects with no MEP response in the affected side had lower LEFM scores in both TA and SO and they were more likely to have difficulty with walking.

**Figure 2:**
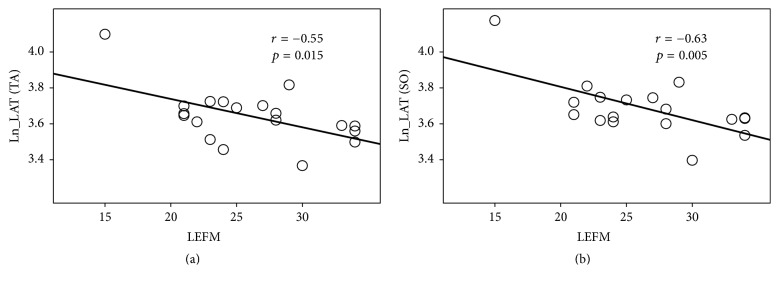
Plot of latency versus LEFM for SO and TA (Ln_LAT versus LEFM score). (a) Ln_LAT at TA. (b) Ln_LAT at SO.

As shown in Figure 2, latency was negatively related to LEFM score.

## 4. Discussion

Outcome measures quantifying poststroke LE impairments are vital to prescribing and gauging response to rehabilitation. To move toward the possibility of applying TMS as an adjunctive or stand-alone LE outcome measurement technique, the primary study objective was to examine associations among MEPs (specifically amplitude and latency) with LE impairment and with gait. Our data suggested that MEP latency is associated with both LE impairment and gait, whereas MEP amplitude was not associated with either metric. This may suggest that a less impaired subject would take a shorter amount of time to reach the amplitude peak, whereas MEPs in a more impaired subject would take a greater amount of time to reach such a peak. Introduced in 1975, the FM is the most established, and one of the most frequently used, instruments in neurorehabilitation. The high degree of agreement between MEP latency and this well-recognized measure supports the precept of using MEP latency as a surrogate LE outcome, per our overall objective. In contrast, the negative findings with regard to MEP amplitude were somewhat unsurprising given that MEP amplitudes have been found unreliable in smaller studies of healthy subjects [32] and in the lesioned hemispheres of chronic stroke subjects when the LE was stimulated [33]. Our results were also unsurprising given the nature of the measures to which MEP outcomes were being compared. As stated earlier, gait velocity can be confounded by a number of extraneous factors such as the patient's fear of falling, strength, and range of motion of the affected extremity. In contrast, LE impairment is a rather pure measure in that it mostly reflects isolated movement of the extremity at targeted joints and is relatively less subject to extraneous factors. One would expect that this movement-based measure would be more closely associated with the activation of the networks modulating this movement. Although convincing and well founded, our findings with regard to MEPs need to be confirmed in a larger sample of subjects.

Our results also showed a direct relationship between the presence of MEPs and impairment as measured by the FM. Corroborating findings have been reported in other studies using nonnavigated TMS techniques. Most notably, Hendricks and colleagues [18] also examined the relationship between the presence of TA MEPs to LE FM scores in subacute stroke, reporting a positive odds ratio of 18 when MEPs were elicited. Although the result was similar to ours, the difference in the magnitude of the results between that study and ours may be related to the subjects' chronicity. Other prospective studies have similarly demonstrated that the presence of MEPs is predictive of LE movement, function [34], and dependence [35], providing additional support for the findings reported herein and impetus for their clinical implementation. While data from the first aim speak to the use of MEP latency as a viable outcome measure, findings from this second aim suggest that the presence of MEPs may offer prognostic value in terms of LE impairment. Taken together, these data provide credence to the notion that MEPs, measured as either the presence of a MEP or by MEP latency, could be coadministered with (or used instead of) impairment-based measures for the LE.

Although not a primary study aim, we also monitored the rate of MEP response in the less affected versus affected brain hemispheres. In the less affected hemisphere, MEP responses were obtained in 88.6% and 85.7% of subjects in the TA and SO, respectively. In contrast, MEP responses were obtained in 54.3% and 51.4% of subjects in the TA and SO, respectively, in the affected hemisphere. These differences were also unsurprising given outcomes of previous work comparing MEPs in the two hemispheres [33]. From a mechanistic standpoint, the differences between the affected and unaffected hemispheres are explainable, given that direct damage to cortical and subcortical neurons would be expected to cause less neuronal firing and, thus, smaller MEP amplitudes on the affected side. Similarly, longer latencies in the affected hemisphere would also be likely to occur due to a decrease in the amount of descending volleys with a subsequently longer period of time required to bring alpha motor neurons to firing threshold.

Stimulating the LE motor cortex accurately is a technically difficult undertaking. By the nature of its location on the homunculus, the LE representation is challenging to reach with a stimulation coil. Navigated TMS may, thus, be advantageous in identifying and stimulating the LE representation, possibly leading to a higher response rate than nonnavigated stimulation and enhanced ability to collect valuable prognostic and outcome data. Our data on responses in the affected and unaffected hemispheres provide preliminary support for this assertion: whereas a recent study using nonnavigated TMS in chronic stroke [36] reported that only 21% of subjects exhibited TA MEPs in the affected hemisphere, ours showed a substantially higher proportion of subjects using navigated TMS. Admittedly, though, this assertion still needs to be confirmed with larger samples. Future researchers may also wish to examine the associations of the MEPs of other LE muscles with impairment and velocity.

## 5. Conclusion

This constitutes the largest poststroke LE MEP study to date, the largest LE study to use navigated TMS, the first to examine associations between LE MEPs and established LE outcomes, and the first to report the parameters of the soleus muscle. The sample size was relatively small, which constitutes a possible study limitation. However this limitation is mitigated by the fact that the sample was well defined (due to relatively stringent study criteria). The contaminating effects of concurrent therapies or medications were taken into account by the study criteria. Moreover, subjects were in the chronic stage, meaning that no spontaneous recovery was occurring. Given these factors and the corroborating results of other studies, it is likely that our findings are valid, and they lay the basis for future work, including interventional studies that examine how MEPs are modulated by active therapy conditions and how these changes conspire with other outcomes to increase independence and quality of life.

## Figures and Tables

**Table 1:** Summary of demographics and baseline characteristics (*N* = 35).

Variable	Category	Statistics
Age^†^		61.6 ± 8.2
Gender	Male^‡^	23 (65.7%)
Time after stroke (months)^†^		36 (2,332)^$^
Dominant_hand	Right^‡^	23 (64.7%)
Affected_side	Right^‡^	19 (54.3%)
Gait speed (cm/sec) without AFO (self-selected)	Able^‡*∗*^	26 (74.3%)
	Speed^†^ (*n* = 26)	81.6 ± 39.8
Gait speed (cm/sec) without AFO (fast)	Able^‡*∗*^	25 (71.4%)
	Speed^†^ (*n* = 25)	109.2 ± 49.5
Difficulty to walk	Yes^‡*∗∗*^	16 (45.7%)
LEFM total^†^		23.5 ± 5.9

^†^Values in cells are median (range).

^‡^Values in cells are frequency (in %).

^*∗*^“Able” to walk without AFO was self reported. Subjects were asked if they felt comfortable walking without the AFO. Gait speed without AFO was tested only for subjects who reported they felt comfortable (*n* = 26 were able to walk without AFO at self selected and *n* = 25 at fast speed).

^*∗∗*^Difficulty to Walk is defined as “Yes” if a person reported not able to walk at either self or fast speed, or was observed below 60 at self-speed or below 80 at fast speed.

LEFM = Lower extremity Fugl Meyer

^$^Value in cell is median (min, max).

**Table 2:** TMS parameters on affected and healthy sides.

Variable	Affected side	Healthy side	*p* value
*TA*			
MEP^†^	19 (54.3%)	31 (88.6%)	0.003
Ln_AMP^‡^	4.85 ± 0.13 (4.87 ± 0.16)	5.38 ± 0.14 (5.39 ± 0.13)	0.035 (0.009)
Ln_LAT^‡^	3.64 ± 0.04 (3.63 ± 0.03)	3.54 ± 0.02 (3.53 ± 0.02)	0.011 (0.014)
*SO*			
MEP^†^	18 (51.4%)	30 (85.7%)	0.001
Ln_AMP^‡^	5.07 ± 0.18 (5.05 ± 0.17)	5.32 ± 0.13 (5.31 ± 0.14)	0.274 (0.214)
Ln_LAT^‡^	3.69 ± 0.04 (3.68 ± 0.03)	3.57 ± 0.02 (3.56 ± 0.02)	0.028 (0.002)

^†^Values in cells are frequency (in %); the *p* value is from McNemar's test.

^‡^Values in cells are mean ± standard error (SE) of log-transformed variables, based upon MEP response patients only. Values in parentheses are mean ± SE from multivariate mixed effect models after adjusting for age, gender, and duration of stroke.

**Table 3:** LEFM and difficulty to walk versus MEP response.

Variable	TA					SO				
	MEP response		Non-MEP response		*p*	MEP response		Non-MEP response		*p*
	*N*	Statistics^†^	*N*	Statistics^†^		*N*	Statistics^†^	*N*	Statistics^†^	
LEFM_Total	19	26.45 ± 1.17 (26.80 ± 1.15)	16	20.31 ± 1.30 (20.96 ± 1.31)	0.002 (0.002)	18	26.74 ± 1.18 (26.99 ± 1.16)	17	20.35 ± 1.24 (21.02 ± 1.27)	0.001 (0.001)
Difficulty to walk	19	26.3%	16	68.8%	0.012	18	22.2%	17	70.6%	0.004
Age	19	61.45 ± 2.22	16	62.13 ± 1.38	0.735	18	61.95 ± 2.28	17	61.53 ± 1.43	0.962

^†^Values in cells are mean ± SE for numerical variables and percentage for the binary variable. Values in parentheses are mean ± SE from multivariate fixed effect models after adjusting for age, gender, and duration of stroke.
